# Effectiveness and Cost-Effectiveness of the Tankevirus and Grubl Mental Health Apps Compared With a Placebo App—Mental Health Intervention With Digital Applications (MIND-APP): Protocol for a Randomized Controlled Researcher-Blinded Trial

**DOI:** 10.2196/84096

**Published:** 2026-08-10

**Authors:** Kim Rand, Julia Menichetti, Torbjørn Wisløff, Hanne Helene Brorson

**Affiliations:** 1 Health Services Research Unit Akershus University Hospital Lørenskog Norway; 2 Maths in Health BV Klimmen The Netherlands; 3 Brorson & Sande AS Oslo Norway

**Keywords:** anxiety, cognitive behavioral therapy, cost-effectiveness, depression, digital health, metacognitive therapy, Norway, QALYs, quality-adjusted life years, randomized controlled trial, self-help, telephone app

## Abstract

**Background:**

Anxiety and depression impose substantial clinical and economic burdens worldwide, with high prevalence, impaired functioning, and elevated health care costs. Digital self-help interventions offer scalable and potentially cost-effective strategies; however, evidence from rigorously controlled economic evaluations remains sparse.

**Objective:**

This trial aims to evaluate the effectiveness and cost-effectiveness of 2 Norwegian mental health apps: Tankevirus (cognitive behavioral therapy–based) and Grubl (metacognitive therapy–based), compared with a digital placebo in reducing anxiety and depression symptoms, improving health-related quality of life, and generating quality-adjusted life years.

**Methods:**

The Mental Health Intervention With Digital Applications (MIND-APP) trial is a 3-arm randomized controlled trial (1:1:1 allocation) conducted fully remotely via a bespoke smartphone research platform. A total of 1000 Norwegian residents aged 16 years or older with mild to moderate symptoms of anxiety and/or depression will be recruited through national digital outreach. Coprimary outcomes are changes in anxiety (Generalized Anxiety Disorder-7) and depression (Patient Health Questionnaire-9) scores from baseline to postintervention (2-4 weeks). Secondary outcomes include health-related quality of life (EQ-5D-5L), quality-adjusted life years accrued over 6 months, functional impairment (Work and Social Adjustment Scale), health care resource use, and adverse events. Incremental cost-effectiveness ratios for Tankevirus and Grubl relative to placebo will be estimated from the perspective of public health services.

**Results:**

Funding was secured in April 2025, with ethical approvals, licensing, and app development planned through 2026. Recruitment will commence in 2027, with follow-up through 2027 and early 2028. An extension of the timetable has been approved by the funding agent to allow inclusion of an updated version of the Tankevirus app, which will be ready for testing around May 2027. Results are expected to be published in autumn 2028 and will provide robust evidence on the clinical and economic value of scalable app-based interventions for common mental health disorders.

**Conclusions:**

This trial will be among the first large-scale registered reports to combine rigorous clinical and economic evaluation of digital mental health interventions. Findings will inform health policy and resource allocation by determining whether low-cost, app-based programs represent cost-effective solutions for reducing the burden of anxiety and depression.

**Trial Registration:**

ClinicalTrials.gov NCT07627204; https://clinicaltrials.gov/study/NCT07627204

**International Registered Report Identifier (IRRID):**

PRR1-10.2196/84096

## Introduction

### Background and Rationale

Mental health disorders represent a growing burden globally and in Norway, with substantial unmet treatment needs. App-based self-help interventions offer scalable, low-cost options for early intervention.

Mental health disorders, particularly anxiety and depression, represent a significant global burden with substantial economic and social consequences. Depression and anxiety are highly prevalent and disabling, resulting in enormous human suffering and lost economic output [1]. In Norway specifically, these disorders affect a considerable portion of the population, with prevalence rates of 21.5% in women and 11.5% in men for depression, anxiety, or somatoform disorders [2]. The incidence has been rising significantly, increasing from 3.3 to 12.8 per 1000 person-years between 1930 and 1991 [2].

These conditions severely impact work participation, with mental health problems accounting for 16.8% of all long-term sick leave incidences and 31.5% of all refunded sick days in Norway [3]. They are also robust predictors of disability pension awards, even when excluding pensions specifically awarded for mental disorders [4]. However, evidence-based interventions such as Norway’s Prompt Mental Health Care show promise in improving both symptoms and functional outcomes [5].

The economic and societal costs associated with anxiety and depression are profound. Direct costs include increased health care service use, general practitioner consultations, and expenditures for medications and therapy. Indirect costs include lost workdays, disability benefits, and decreased work performance. On a global scale, the World Health Organization (WHO) estimates that mental health disorders accounted for 16% of disability-adjusted life years in 2019, with an economic burden estimated at about US $5 trillion [6], with the majority of these costs typically associated with anxiety and depression [7]. The human burden, including reduced quality of life, increased morbidity, and elevated suicide risk underscores the urgent need for effective intervention strategies.

Addressing these needs, Norway has prioritized mental health in its public health agenda, focusing on early intervention, destigmatization, and equitable access to care [8]. The development and evaluation of scalable self-help apps have the potential to offer accessible, tools to ameliorate symptoms or prevent escalation into more severe disorders; however, evidence is sparse [9].

Internationally, the United Nations Sustainable Development Goals and the WHO Comprehensive Mental Health Action Plan 2013-2030 call for a significant reduction in the burden of mental health disorders [10]. The emphasis is on integrating mental health into primary care, investing in research and digital innovation, and promoting mental health literacy. Both Norway and the global community recognize that meeting these goals requires coordinated action across health systems, communities, and policy frameworks, with a commitment to improving the lives of those living with anxiety and depression.

Digital interventions have shown promise in reducing symptoms of common mental disorders [11], with greater effect sizes observed in apps applying cognitive behavioral therapy (CBT) principles. Tankevirus and Grubl (literally translated as Thought Viruses and Overthinking) are 2 widely used Norwegian apps based on cognitive and metacognitive therapeutic principles, respectively.

CBT addresses reciprocal relationships between thoughts, feelings, and behaviors. By identifying and restructuring maladaptive cognitions, framed as “thought viruses” in the Tankevirus app, CBT-based interventions framed as “psychological vitamins” aim to interrupt the negative cycles that maintain and escalate anxiety and depression. App-based CBT delivery has demonstrated effect sizes (Cohen *d*) of 0.3 to 0.5 on anxiety and depression in meta-analyses, although evidence quality and effect sizes vary considerably.

Metacognitive therapy (MCT) targets distinct mechanisms described under the Cognitive Attentional Syndrome: metacognitive beliefs about the uncontrollability or usefulness of worry and rumination, and threat monitoring. MCT techniques, including detached mindfulness and attention training, aim to reduce perseverative thinking and disrupt maladaptive attentional strategies, rather than alter specific thought content. Grubl operationalizes these MCT principles for app-based self-help. While therapist-delivered MCT has a strong evidence base, no randomized controlled trials (RCTs) have, to our knowledge, evaluated a standalone self-help app based on MCT.

Based on self-reporting from users of Tankevirus and Grubl, more than 70% of users show improvements in anxiety, depression, somatic symptoms, and health-related quality of life (HRQoL) after 1 month. However, the extent to which this improvement can be attributed to the apps remains uncertain in the absence of a suitable control. This study addresses this evidence gap by evaluating these apps using a blinded RCT design.

A key methodological challenge in digital mental health research is the absence of credible control conditions. Without a matched control, symptom improvements cannot be attributed to specific therapeutic content rather than nonspecific factors such as self-monitoring, expectancy of benefit, digital engagement, or natural fluctuation. A “digital placebo,” an app matching structural features of the active interventions such as duration, format, push notifications, and daily content while delivering content without known therapeutic mechanisms, provides the rigorous comparator that prior evaluations of Tankevirus and Grubl have lacked.

A waitlist-based comparator was considered but will not be used, as there is indication that being assigned to a waitlist has been found to potentially function as a nocebo [12]. Of particular concern with the provision of digital self-help apps is the lack of a plausible reason for why participants should be assigned to a waitlist beyond a wish to capture a baseline, as capacity limitations are unlikely with such apps.

Despite the high prevalence and economic burden of anxiety and depression, evidence on the cost-effectiveness of digital self-help interventions remains sparse. A cost-effectiveness analysis requires standardized health outcome measurement (here: quality-adjusted life years [QALYs] from EQ-5D-5L) combined with resource use data. App-based interventions have low marginal delivery costs, creating conditions potentially favorable for cost-effectiveness relative to therapist-delivered care when delivered at scale. However, rigorous economic evaluations of mental health apps in controlled trials remain rare.

Both Tankevirus and Grubl are widely used in Norway but address anxiety and depression through distinct therapeutic frameworks, CBT and MCT, respectively. Including both in a single trial enables independent evaluation of each against a matched digital placebo, providing the first placebo-controlled evidence for either app. The trial is designed and powered for separate formal statistical comparison between Tankevirus and placebo and between Grubl and placebo, but not for a comparison between Tankevirus and Grubl. Determining the required power for a direct comparison of the 2 apps would require assumptions regarding their relative efficacy, which we do not presently have. The trial design maximizes the practical and policy-relevant output from a single trial.

### Objectives and Hypotheses

Primary objective: to evaluate the effectiveness of Tankevirus and Grubl in reducing symptoms of anxiety compared with a digital placebo condition following intervention completion (14 days to 4 weeks postbaseline, depending on app use and response delays).

Secondary objectives include evaluating the effectiveness of Tankevirus and Grubl in reducing symptoms of depression compared with a digital placebo condition following treatment completion (approximately 4 weeks); efficacy in reducing anxiety and depression symptoms at 3- and 6-month follow up; improving HRQoL between baseline and treatment completion, 3 months, and 6 months; providing greater accrual of QALYs than placebo between baseline and treatment completion, 3-month follow up, and 6-month follow-up; and being cost-effective in terms of incremental cost-effectiveness ratio (ICER) compared with the placebo condition when accounting for the cost of the apps and costs related to health care resource use during the trial observation period.

The primary hypothesis is that use of Tankevirus or Grubl will result in a greater reduction in symptoms of anxiety as measured using Generalized Anxiety Disorder-7 (GAD-7) and depression as measured using Patient Health Questionnaire-9 (PHQ-9) than a digital placebo condition. We also hypothesize that Tankevirus and Grubl will provide greater improvement in HRQoL, greater accrual of QALYs, and a favorable ICER over placebo when accounting for the cost of the apps and self-reported health care resource use.

## Methods

### Standard Protocol Items

This protocol is designed to comply with the SPIRIT (Standard Protocol Items: Recommendations for Interventional Trials) 2025 guidelines. The development of this protocol has been inspired by protocols for previously conducted RCTs for mobile phone apps targeting mental health issues, in particular the Healthy Campus Trial covering an RCT for CBT apps promoting mental health in university students [13] and the DEFINE trial covering use of a CBT-based app to alleviate the symptom burden related to tinnitus [14], in which part of the study team was involved. The completed SPIRIT 2025 checklist is available in Multimedia Appendix 1.

### Aim, Design, and Setting of the Study

The Mental Health Intervention With Digital Applications (MIND-APP) is an open-label, prospective parallel-design, randomized controlled clinical trial. The trial is designed to evaluate the effectiveness and cost-effectiveness of the Tankevirus and Grubl self-help mobile phone apps compared with a vehicle control app in patients aged 16 years or older with mild to moderate anxiety and/or depression. MIND-APP is to be entirely remotely delivered via a bespoke research app made available on the Apple Store for iOS devices and the Play Store for Android devices. Potential participants will be recruited through social media and through mental health patient organizations, with links to a trial web page and the MIND-APP RCT application. Following registration and explicit informed consent, potential participants are administered a screening questionnaire to determine whether they have mild to moderate anxiety and/or depression. With this approach, the trial aims to achieve a wide coverage of the Norwegian general population with mild to moderate anxiety and/or depression.

The trial is registered at ClinicalTrials.gov (NCT07627204). This manuscript describes protocol version 1.0, dated April 10, 2026. Reporting of the protocol is according to SPIRIT guidelines.

### Inclusion and Exclusion Criteria

The target population comprises Norwegian-speaking adults or adolescents aged 16 years or older who experience mild to moderately severe symptoms of anxiety and/or depression and are seeking digital mental health support. Eligible participants must not have previously used Grubl or Tankevirus. Inclusion and exclusion criteria are provided in Textbox 1.

**Inclusion and exclusion criteria.**

**Inclusion criteria**

- Adults and adolescents aged 16 years or older (16 is the minimum age of consent without parental approval for participation in research in Norway. Inclusion of individuals aged younger than 18 years will be highlighted in the ethical review.)
- Residing in Norway
- Access to a compatible smartphone (iOS or Android)
- Reporting mild to moderately severe symptoms of anxiety and/or depression (Patient Health Questionnaire-9 [PHQ-9] score ≥5 and/or Generalized Anxiety Disorder-7 [GAD-7] ≥5)
- Able to read Norwegian and use Norwegian to a sufficient level
- Able and willing to provide consent for the study prior to participation

**Exclusion criteria (based on self-reporting)**

- PHQ-9 score ≥20 (severe depression)
- Active suicidal ideation (PHQ-9 item 9 score ≥2)
- Have ever required hospitalization for mental illness or are currently taking antipsychotic or other psychotropic medications
- Current engagement in psychological treatment
- Report having used Tankevirus or Grubl apps in the past

### Recruitment

Participants will be provided with a link to a trial website containing information on the objective and structure of the trial, as well as the planned compensation for participation. They will also be provided with links to the MIND-APP app in the Google Play Store and iOS App Store.

Platforms for outreach include

- Social media advertising (Facebook, Instagram, and other platforms)
- University student health services
- Primary care clinics (informational materials only)

In primary care settings, informational posters and flyers will be placed in waiting rooms to direct self-referring patients to the trial website; no active general practitioner screening or referral is involved.

- Mental health advocacy organizations
- Patient community advertising

### Randomization and Blinding

Following registration and completion of the screening questionnaires, eligible participants are automatically randomized within the MIND-APP app. Table 1 outlines the time points of enrollment, interventions, and assessments.

**Table 1:** MIND-APP trial schedule of enrollment, interventions, and assessments.

Time point			Study period					
			Enrollment (T0)		Postintervention follow-up^a^ (approximately 4 weeks)		3 months	6 months
**Administrative procedures**								
	Eligibility screening	✓						
	Informed consent	✓						
	Treatment allocation	✓						
	Compensation/voucher			✓				✓
**Interventions**								
	Tankevirus	✓						
	Grubl	✓		✓				
	Placebo	✓		✓				
**Assessments**								
	PHQ-9^b^	✓		✓		✓		✓
	GAD-7^c^	✓		✓		✓		✓
	EQ-5D-5L	✓		✓		✓		✓
	WSAS^d^	✓		✓		✓		✓
	CSQ-8^e^			✓				
	SUS^f^			✓				
	Co-treatment check	✓		✓		✓		✓
	Resource use	✓		✓		✓		✓

^a^Postintervention follow-up was completed after program completion or after a maximum of 4 weeks.

^b^PHQ-9: Patient Health Questionnaire-9.

^c^GAD-7: Generalized Anxiety Disorder-7.

^d^WSAS: Work and Social Adjustment Scale.

^e^CSQ-8: Client Satisfaction Questionnaire-8.

^f^SUS: System Usability Scale.

Participants will be randomized in a 1:1:1 ratio to Tankevirus, Grubl, or the placebo app. The app backend will automatically handle randomization, which, following a burn-in of 100 participants, will be conducted using covariate-adaptive minimization with a biased coin [15].

Stratification factors are sex, age group (18-30 years, 31-50 years, and ≥51 years), and baseline severity (above/below GAD-7 score 10 or PHQ-9 score 10).

Allocation concealment is maintained by the centralized MIND-APP RCT platform, which stores arm assignments in a database partition inaccessible to study team members prior to data lock. Analyst blinding is maintained by removing arm identifiers from the analysis dataset until all primary end point analyses are locked; unblinding is performed by the platform administrator upon confirmation of analytical lock.

Following randomization, no attempts will be made to mask the allocated app from the participants: Tankevirus and Grubl are both branded and have distinct visual styles. However, participants will not be provided with information on which options they were not randomized to.

All recorded data will be stored in a way allowing the research team to be blinded to which allocation number is tied to which appl until all comparative analyses have been concluded; the key will only be revealed once the full research team agrees that the protocolized analyses are complete.

An automatic audit trail is maintained for all data access and modifications within the trial database, as required by good clinical practice.

The study will follow a fully remote design, with all procedures conducted via smartphone (initial information through the website can be accessed in any web browser). Allocation will be blinded to researchers and statisticians. Participants will be aware of their assigned app but will not be informed about the contents of the alternative arms.

### Interventions

#### Overview

No medication is administered within the study, nor are any medications prohibited.

Participants are also prohibited from using other self-help mental health smartphone apps during the 4-week intervention period. Adherence to this rule will be assessed at each follow-up time point via a dedicated question in the app. Concomitant psychological therapy is prohibited during the 4-week intervention period but is permitted during the 3- and 6-month follow-up phases; its use will be recorded as a potential covariate in sensitivity analyses.

Tankevirus and Grubl are 2 structurally similar self-help apps with different visual design, different lengths, and different contents. Shared characteristics include:

- Onboarding with baseline measurement of mental health with feedback
- Psychoeducational videos
- A set of shorter videos and tasks delivered 1 each day following the introductory videos
- Mental health measurement following completion, with feedback on self-reported change
- Long-term follow-up measurement at 3 and 6 months
- Automatic reminders (daily push notifications)
- Program progress tracking
- Options to rewatch elements of particular interest

The regular versions of Tankevirus and Grubl use a set of standardized questionnaires used to track mental health, specifically the Patient Health Questionnaire-4 (PHQ-4) inventory, which comprises 2 questions from the PHQ-9, covering depression, and 2 questions from the GAD-7, covering anxiety; the Somatic Symptom Scale 8-item (SSS-8); and a visual analogue scale inspired by the one used in the EQ-5D family of instruments. In the MIND-APP implementation, these are substituted by the longer PHQ-9 and GAD-7 instruments, and the full EQ-5D-5L.

The placebo condition will be designed to mimic these structural elements, with the exception of the content elements, as described below

#### Tankevirus

**Figure 1:**
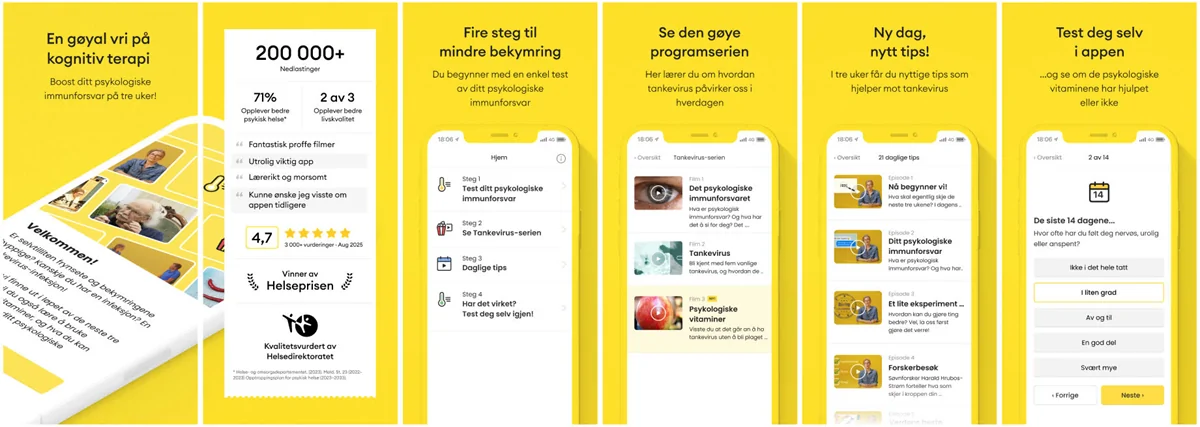
Tankevirus flow and screenshots (in Norwegian).

Tankevirus is a novel take on CBT that leverages accessible metaphors to explain how resilience can be built by addressing essential needs such as sleep (the psychological immune system), recognizing negative thought patterns (thought viruses), and developing healthier coping mechanisms (psychological vitamins).The metaphors are designed to help users identify and restructure maladaptive thought patterns in a way that is engaging and easy to understand, particularly for individuals with low health literacy or high psychological distress. Tankevirus targets mild to moderate anxiety and depressive symptoms and has been widely disseminated in Norway, with approximately 4% of the population having used the current version of the app. The app features psychoeducational and skill-building videos. Figure 1 displays screenshots of the app as displayed in the Play Store (in Norwegian).

A new version of the Tankevirus app is under development and will be ready for deployment in late 2026. This RCT will use the new version of the app, which is expected to replace the existing one subsequent to the trial. The duration of the new version will be approximately 14 days and include improvements to videos, tasks, and display elements based on user feedback and research received after the development of the first version of the app.

#### Grubl

**Figure 2:**
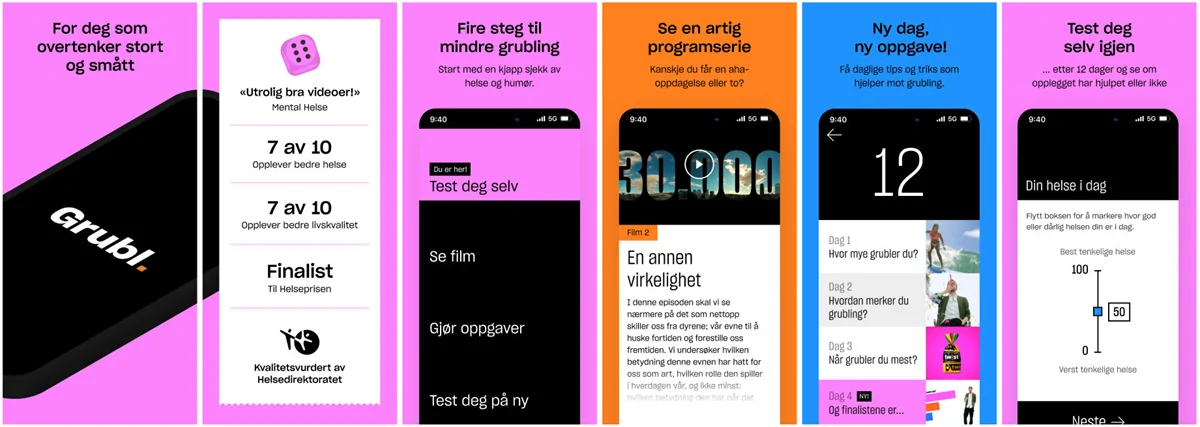
Grubl flow and screenshots (in Norwegian).

Grubl is a 12-day app-based self-help intervention based on MCT. It was developed in collaboration with general practitioners in Norway to meet the needs of patients presenting with excessive worry and rumination, issues estimated to account for around 20% of primary care consultations. Grubl targets users experiencing elevated stress and somatic symptoms, often linked to persistent rumination and worrying. The intervention provides psychoeducation on the mental and physical impact of overthinking and focuses on detached mindfulness, attention training techniques, and challenging metacognitions about worry and rumination. Figure 2 displays screenshots of the app as displayed in the Play Store (in Norwegian).

#### Placebo Condition

To enable valid comparison between groups, a digital control condition will be developed. The control condition will be designed to fulfill the following criteria:

- Minimize danger of harm to users
- App contents should convey no or minimal potential benefit in terms of alleviation of symptoms of anxiety and depression
- Have face validity to provide similar levels of user expectancy regarding potential efficacy to the active treatment arms
- Similar levels of user engagement and retention in order to limit dropout and preserve statistical power

The control app will closely mirror the active interventions (Tankevirus and Grubl) in all nonspecific aspects of delivery and user experience. Specifically, the following features will be matched: duration, push notification frequency, visual design and navigation, daily content format, progress tracking mechanisms (eg, session counters or progress bars), interface feedback (eg, simple acknowledgements of completion), and assessment schedule and format. Onboarding, consent procedures, and in-app interactions will also be aligned across arms. This design ensures that any observed differences in outcomes are unlikely to be attributable to expectancy bias, delivery format, user experience, or perceived program structure.

We explicitly intend to err on the side of potentially providing a control condition involving some minimum level of treatment effect if that is required to achieve the other goals**.** This implies that the “placebo” condition may not reflect ideal placebo but rather an intervention with no or limited intended treatment effect. This deviation from an ideal placebo will bias comparative effectiveness results against the active treatments, which is a conservative approach.

**Figure 3:**
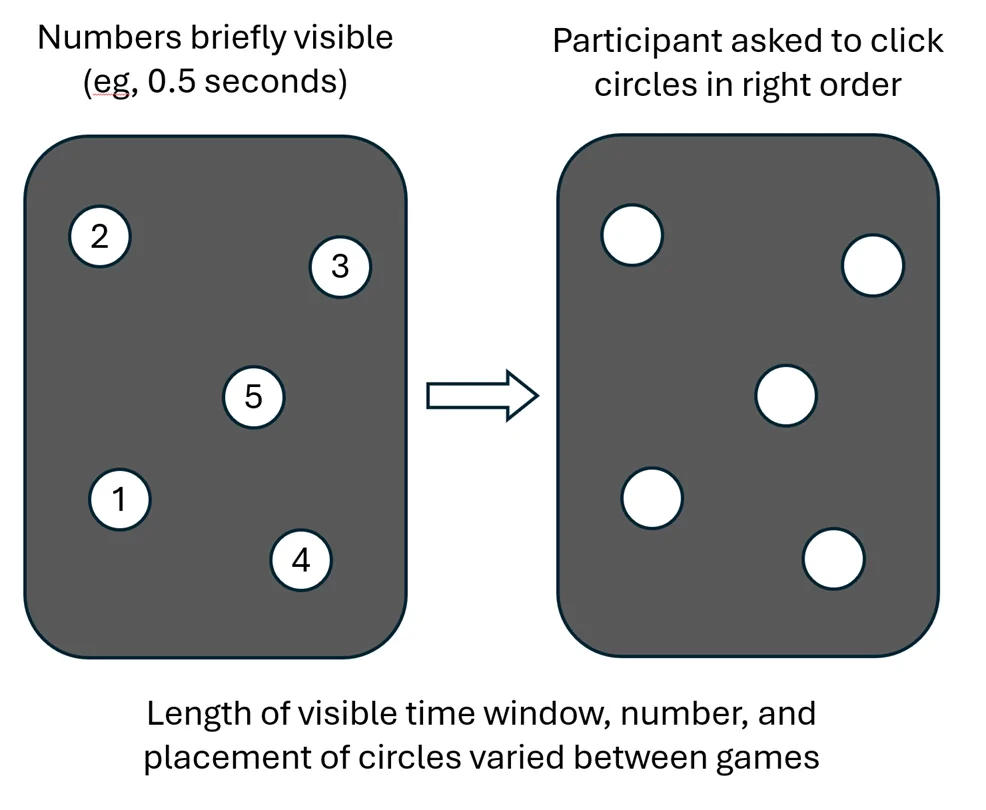
Example of attention task in placebo arm.

The content of the placebo app will be short introductory videos followed by daily exercises in the form of small concentration/attention games. The introductory videos will explain that attention training has been demonstrated to ameliorate anxiety and depression (eg, the literature on Attention Training Technique [16-18]) and that the intervention consists of small attention/concentration games that should be completed in a few minutes each day. A mock-up of one such game is given in Figure 3. While research suggests that attention training is indeed helpful in reducing symptoms of anxiety and/or depression, the games and the volumes of training provided are unlikely to convey any meaningful therapeutic effect. As such, this is a sham intervention, ideally with sufficient face validity to be convincing to participants. Importantly, spending a few minutes daily over a couple of weeks completing these attention/concentration games is considered harmless to study participants.

Prior to full rollout, the control app will be pilot-tested to assess perceived credibility and engagement, and to confirm the absence of symptom change in small-scale user samples.

A detailed description of the control condition will be developed and included in Multimedia Appendix 2 (currently a placeholder) and will be finalized and dated prior to initiation of data collection.

Participants in the control group will be offered access to the active apps when the study is completed and data collection has been closed.

### Assessments

All clinical questionnaires will be administered at screening/baseline, at intervention completion (4 weeks), 3 months, and 6 months.

Inclusion of all measures is conditional on approval and/or licensing for the intended purpose.

#### Outcome Measurement Instruments

The GAD-7 is a 7-item self-report scale developed to assess the severity of generalized anxiety disorder symptoms [19]. It is widely used as a brief screening and outcome measure for anxiety in clinical practice and research [20,21]. Measured at baseline, postintervention, and at 3- and 6-month follow-up. Items are scored 0 to 3 (total score 0 to 21). Cutoffs: 5 (mild), 10 (moderate), and 15 (severe). Excellent reliability (Cronbach α≥0.92).

The PHQ-9 is a 9-item self-report instrument designed to assess depressive symptom severity based on *DSM-IV* (*Diagnostic and Statistical Manual of Mental Disorders* [Fourth Edition]) criteria [22]. It is a commonly used tool for depression screening and monitoring treatment outcomes in research and practice [23,24]. Items are scored 0 to 3 (total score 0 to 27). Cutoffs: 5 (mild), 10 (moderate), 15 (moderately severe), and 20 (severe). Measured at baseline, postintervention, and at 3- and 6-month follow-up.

The EQ-5D-5L, developed by the EuroQol Group [25], measures HRQoL across 5 domains (mobility, self-care, usual activities, pain/discomfort, and anxiety/depression) with 5 severity levels. It is extensively used in clinical trials, health economics, and QALY calculations [26]. The Norwegian EQ-5D-5L tariff will be used. Delta QALYs will be calculated using interpolation over the 6-month observation period. The EQ visual analog scale (EQ-VAS; 0-100) captures self-rated health. Utility values for QALY calculation will use the Norwegian EQ-5D-5L value set [27]. Measured at baseline, postintervention, and at 3- and 6-month follow-up.

The Work and Social Adjustment Scale (WSAS) is a 5-item self-report scale developed to measure functional impairment attributable to a specific health problem across work, home management, social and private leisure activities, and relationships [28]. It is frequently used to quantify disability in mental health research and evaluate treatment impact. Scores range from 0 to 40; higher scores indicate greater impairment. A score >20 indicates moderately severe or worse impairment. Measured at baseline, postintervention, and at 3- and 6-month follow-up.

The Client Satisfaction Questionnaire-8 (CSQ-8) is an 8-item self-report tool assessing client satisfaction with health and mental health services. It is widely applied in service evaluation and clinical trials to measure acceptability and perceived benefit of interventions [29]. Administered at postintervention follow-up only.

The System Usability Scale (SUS) [30] is a 10-item Likert-scale questionnaire providing composite app usability score (0-100). The SUS has become an industry standard for app assessment [31]. Administered at postintervention follow-up only.

#### Primary Outcomes

The co-primary outcomes in the trial are (1) the difference in the GAD-7 score for anxiety symptoms between baseline and postintervention for participants with mild to moderately severe self-reported problems with anxiety at baseline (GAD-7 score ≥5), and the difference in the PHQ-9 score for symptoms of depression between baseline and postintervention for participants with mild to moderately severe self-reported problems with depression at baseline (PHQ-9 score ≥5).

#### Secondary Outcomes

The secondary outcomes are (1) change in anxiety symptoms measured by the GAD-7 between baseline and 3 months and 6 months in participants reporting problems at baseline (GAD-7 ≥ 5), (2) change in levels of depressive symptoms as measured using the PHQ-9 for between baseline and 3 months and 6 months in patients reporting problems at baseline (PHQ-9 ≥ 5), and (3) QALY accrual as measured using the EQ-5D-5L, and (4) change in functional impairment as measured using the WSAS.

#### Other Assessments

Other assessments include (1) client satisfaction following completion of the app using the CSQ-8 at postintervention (tertiary outcome); (2) the SUS (tertiary outcome); (3) app engagement metrics (sessions completed, time spent, features used; automatically recorded; tertiary outcome); (4) self-reported use of health services (for use in calculation of costs and as a tertiary outcome); and (5) use of concurrent psychological treatments assessed at each follow-up time point with type and frequency of any concurrent treatment recorded and included as covariates in sensitivity analyses.

### Participant Pathway

**Figure 4:**
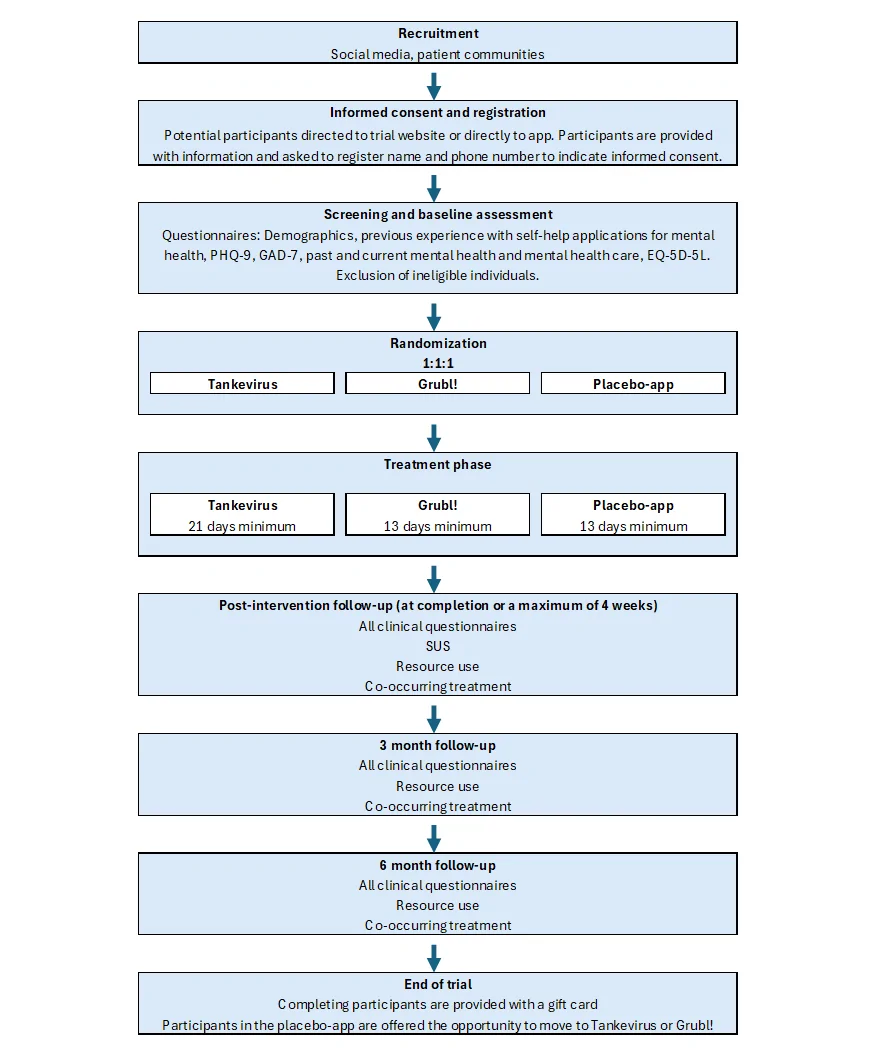
Flowchart showing the patient pathway from recruitment to final follow-up. All timings are measured from the timing of the first “dose,” that is, the first main video of the Tankevirus and Grubl arms or the first main element of the placebo arm. GAD-7: Generalized Anxiety Disorder-7; PHQ-9: Patient Health Questionnaire-9.

The participant pathway is summarized in Figure 4. Potential participants will be guided to a trial website or directly to the iOS/Android MIND-APP app.

Once MIND-APP is installed, participants will be asked to read the provided trial information, after which they will be asked to provide consent by providing their name, age, and phone number. To complete registration, they will be sent an SMS text message with a 1-time code that they enter in the app. As described in the section “Data Management and Protection,” the name and phone number will be stored separately from all other trial data and will be used for the following purposes:

- To ascertain that all participants are real persons
- To verify identity and prevent duplicate registrations

Participants provide their full name and mobile phone number during the registration process. This information is transmitted via HTTPS through the encrypted app backend and stored in a separate database from all trial data. It is used solely for identity verification and compliance with accounting regulations for voucher distribution and is deleted upon completion of data collection.

Participants may request withdrawal and deletion of their data at any point up to the formal end of the trial. After this point, data will have been deidentified and merged into the locked analysis dataset; individual-level deletion will no longer be possible.

The formal end of the trial is defined as the date of the last participant’s last data capture (6-month follow-up assessment).

Following data collection cutoff, all participants are offered access to the 2 active intervention apps (Tankevirus and Grubl).

Financial compensation consists of NOK 500 (approximately US $50), to be provided either as a one-off upon completion of the 6-month follow up or in 2 tranches of NOK 250 (approximately US $25) at completion of the primary follow-up and the 6-month follow-up. As financial compensation can function as a disincentive for some potential participants, all participants will be offered the option to donate to a set of charitable causes as an alternative to receiving gift cards. For participants eligible to receive compensation in the form of gift card vouchers, the name and phone number will be transferred to the Akershus University accounting department to comply with accounting regulations.

A temporary key will be used to tie participant trial data to the name and phone number to allow provision of gift cards at the end of the trial. This link will be deleted as soon as the gift cards have been issued and the trial is completed or as soon as the potential participant is identified as ineligible.

Participants will be informed that they have the option to withdraw from the trial and request deletion of any registered data up until the first event resulting in deletion of the link between the registered phone number and the corresponding trial data, which will occur once they have completed the 6-month follow-up or once 7 months have elapsed since the first use of the app.

### Screening and Baseline Assessment

Following registration and informed consent, potential participants are issued a set of screening questionnaires.

#### Demographics

Demographics include: current country of residence, age, sex, and highest attained educational level using the categories used by Statistics Norway*.* Participants will be excluded if they do not indicate that they live in Norway or if they indicate an age <16 years.

#### Previous Use of Self-Help Apps for Mental Health

A list of mobile phone–based self-help apps available for use in Norway will be provided along with instructions to check any that have been used in the past. A tentative list can be found in Multimedia Appendix 3, which will be updated shortly prior to recruitment. Participants will be excluded if they indicate that they have previously used Tankevirus or Grubl.

#### Current Level of Anxiety and Depression

Current level of anxiety and depression will be measured using the GAD-7 and the PHQ-9. Participants will be excluded if they report a total PHQ-9 score ≥20, indicating severe depression; if they report a score ≥2 on PHQ-9 item 9, indicating active suicidal ideation; or if they report a total score ≤4 on both GAD-7 and PHQ-9, indicating that they do not have at least mild levels of either anxiety or depression.

#### Questions Regarding Current and Past Health Care Use for Mental Health Issues

Participants will be asked whether they have at any point in the past required mental health care of different types, and will be asked to check any in a list of different types of health services they have used in the past, including follow-up from general practitioner; having seen a psychologist or psychiatrist; and having required hospitalization.

Participants will be asked whether they currently receive any mental health support and will be asked to check any that are relevant from a list. Participants will be excluded if they indicate that they are currently being treated for mental health issues or if they indicate that they have previously required hospitalization for mental health issues.

Excluded individuals will be thanked for their interest and informed of the reason for exclusion. Participants indicating severe depression (PHQ-9 ≥20) or responding with a score of ≥2 on PHQ-9 item 9, suggesting suicidal ideation, will also be provided with information on where they can seek help. Draft information to be provided is available in Multimedia Appendix 4. Individuals excluded based on other criteria will be provided with links to public information on mental health and links to the regular Tankevirus and Grubl apps. All excluded individuals will be informed that all the information provided will be deleted once recruitment is complete.

Given the financial incentive provided, there is a risk of individuals attempting duplicate participation. While this cannot fully be ruled out, the risk is limited by use of phone number and full name at registration.

Eligible participants will be asked to complete remaining baseline questionnaires before randomization.

Participants are informed that they will have the option to move on to another treatment option following closure of the data collection.

#### Randomization

Participants are randomized in a 1:1:1 ratio to Tankevirus, Grubl, or the placebo condition.

#### Treatment Phase

Participants follow the Tankevirus, Grubl, or placebo app for the full duration, with automatic reminders in the form of notifications.

#### Postintervention Follow-Up

Upon completion of the last program element or after 4 weeks, participants will be invited to respond to the postintervention follow-up. This comprises all clinical questionnaires (PHQ-9, GAD-7, EQ-5D-5L, and WSAS), retrospective health care resource use over the last 4 weeks, questions about co-occurring treatment, SUS, and the CSQ-8.

Participants who have not submitted any outcome assessment within 2 weeks of the scheduled assessment date, despite automated push notification reminders, will be considered lost to follow-up for that time point. This rule applies at postintervention and at 3- and 6-month follow-up.

#### 3-Month Follow-Up

Participants are administered all clinical questionnaires (PHQ-9, GAD-7, EQ-5D-5L, and WSAS), retrospective health care resource use since the postintervention follow up, and questions about co-occurring treatment.

#### 6-Month Follow-Up

Participants are administered all clinical questionnaires (PHQ-9, GAD-7, EQ-5D-5L, and WSAS), retrospective health care resource use since the 3-month follow-up, and questions about co-occurring treatment.

#### End of Trial

Completing participants are provided with an electronic gift card voucher valued at NOK 500 (approximately US $50). This is tentative; another option is to provide each participant with 2 gift cards valued at NOK 250 (approximately US $25); 1 following completion of the postintervention follow-up, and 1 following the 6-month follow-up. The completion criterion here is exclusively linked to response to questionnaires and not to the use or completion of app content.

### Safety Considerations

Use of MIND-APP is considered safe for users. Tankevirus and Grubl provide videos with psychoeducational contents, and the placebo app will include a set of short attention/concentration games to be done in a few minutes each day. Identifiable information is stored separately from recorded trial data. However, we cannot conclusively exclude the possibility that study participants experience discomfort while using the apps.

While the intervention type involved in MIND-APP is unlikely to be associated with most types of adverse events, monitoring is conducted as per clinical RCT conventions.

Adverse events are defined as any untoward medical occurrence in a participant to whom a medicinal intervention has been administered, including occurrences that are not necessarily caused by or related to that product. Serious adverse events are defined as any untoward medical occurrence that: results in death, is life-threatening, requires inpatient hospitalization or prolongation of existing hospitalization, results in persistent or significant disability/incapacity, or consists of a congenital anomaly or birth defect. The term “life-threatening” in the definition of “serious” refers to an event in which the participant was at risk of death at the time of the event; it does not refer to an event that hypothetically might have caused death if it were more severe.

Other “important medical events” may also be considered a serious adverse event when, based on appropriate medical judgement, the event may jeopardize the participant and may require medical or surgical intervention to prevent one of the outcomes listed above.

Participants may report potential adverse events via a dedicated menu option in the app or by contacting the trial team directly by phone or email. Adverse events will be collected systematically from randomization through the 6-month follow-up assessment.

While unlikely for most users, repeated self-monitoring or exposure to sensitive psychoeducational topics might prompt distress, anxiety, sadness, or increased rumination in some participants.

Self-monitoring could lead to heightened symptom awareness, possibly resulting in perceived or actual increases in distress.

Frustration, confusion, or distress could result from app malfunctions, usability challenges, or notification fatigue.

Worsened symptoms will be captured by the recording of symptoms in the trial. In addition, a menu item not present in the regular Tankevirus and Grubl apps will be provided, allowing users to report any discomfort and to optionally complete PHQ-9 and GAD-7 to report their current mental health. Users indicating severe depression (PHQ-9 score ≥20) or reporting score ≥2 on item 9 of PHQ-9, suggesting suicidal ideation, will be provided with information on where they can seek help (Multimedia Appendix 4).

The relationship of each adverse event to the intervention must be determined by a qualified individual according to the following definitions:

- Unrelated: where an event is not considered to be related to the intervention.
- Possibly: although a relationship to the intervention cannot be completely ruled out, the nature of the event, the underlying disease, concomitant medication, or temporal relationship make other explanations possible.
- Probably: the temporal relationship and absence of a more likely explanation suggest the event could be related to the intervention.
- Definitely: the known effects of the intervention, its therapeutics class, or findings from challenge testing suggest that the intervention is the most likely cause.

All adverse events (serious adverse events) labeled possibly, probably, or definitely will be considered related to the intervention.

We do not anticipate that the study interventions (Tankevirus, Grubl, or the placebo app) should result in any adverse events but this section is included in case such events are reported so that they can be considered for causal links to the study. Only adverse events that are clinically judged by the research site monitoring board as being caused by the trial intervention will be reported in accordance with Akershus University Hospital guidelines. Participants will be asked at follow-up whether they have experienced any side effects or medical events while participating in the MIND-APP trial by way of a reporting option in the app. Participants may also report potential serious adverse events via phone or email correspondence with the trial team.

We do not anticipate any serious adverse events due to the digital interventions or data collection.

### Sample Size Calculation

Sample size calculation assumptions were based on self-reported data from the Tankevirus app in Norway combined with information from 2 meta-analyses of mobile apps for mental health [11,32]. The 2 meta-analyses both indicate high variability both in quality of evidence and estimated effect sizes. Assuming low to moderate effects, we assumed Cohen *d* of 0.3. For a single outcome, targeting 80% power and a critical α of .05, regular power analyses indicate a need for 176 participants in each study arm. However, the coprimary end points of the trial and expected correlation between the GAD-7 and the PHQ-9 imply reduced power.

To better reflect these factors, we built a Monte Carlo simulation based on recorded responses to the PHQ-4, which comprises 2 questions from the GAD-7 and 2 questions from the PHQ-9 collected from the existing Tankevirus app. We assume a correlation of approximately 0.7 between the PHQ-9 and the GAD-7. Assuming a distribution of responses taken from these self-report data, targeting 80% power, with a critical α=.05, the analyses suggested that 300 participants per arm are required for comparisons between each active arm and the placebo condition, given the coprimary end points and the observed proportions having problems with either anxiety or depression but not both.

The trial is powered for separate comparison of each app to placebo, but is not powered to allow meaningful comparison of the outcomes between Tankevirus and Grubl. The recruitment target is 1000 participants to account for attrition.

### Data Management and Protection

All trial data will be collected via the MIND-APP framework. The database server will be behind a virtual private network with a firewall, requiring a combination of username, password, and certificate for access. Access to the database system further requires a username and password. An automatic audit trail will log all data access.

Directly identifiable information, including name and phone number, will be recorded at the informed consent stage and will be stored separately from the trial data, encrypted, and stored on servers compliant with Norwegian data protection regulations (GDPR [General Data Protection Regulation] and Norwegian Personal Data Act). Identifiable information will be required for 2 primary purposes:

- To ascertain that all participants are real persons
- To reduce the risk of repeat registrations, both for eligible and ineligible individuals

For participants eligible to receive compensation in the form of gift card vouchers, the name and phone number will be retained to comply with accounting regulations.

The link between the directly identifiable information and trial data will be deleted upon completion of data collection and prior to analysis.

Anonymized data will be retained indefinitely for research purposes.

Study materials and essential documents (trial master file, analysis datasets, and regulatory correspondence) will be archived by Akershus University Hospital for a minimum of 10 years following trial completion, in accordance with Norwegian research regulations.

### Statistical Analysis Plan

#### Statistical Methods

Observed values from the per-protocol population will be used for the primary analysis. Sensitivity analyses including complete cases and an intention-to-treat analysis with imputed values will also be conducted. The RCT app automatically records all use of all app functions with timestamps, and per-protocol compliance will be determined as having viewed the 3 primary videos of the Tankevirus and Grubl arms or having completed the 3 primary units of the placebo arm. Participants with compliance below the per-protocol threshold will be included in the intention-to-treat analysis but not the per-protocol analysis.

The primary analysis will use an analysis of covariance (ANCOVA) framework: the postintervention score will be the dependent variable, treatment arm the primary factor, and baseline score a covariate. This adjusts for regression to the mean and improves precision. Stratification variables used in randomization (sex, age group, and symptom severity) will also be included as covariates.

The trial compares 2 separate treatments with placebo, and the literature is divided on whether testing should be adjusted for multiplicity [33]. To account for the 2 treatment-vs-placebo comparisons while controlling the familywise type I error rate, Dunnett procedure will be applied to the ANCOVA-adjusted (least squares) means, exploiting the positive correlation between the 2 test statistics arising from the shared placebo arm [34].

#### Coprimary Outcomes

The coprimary outcomes will be change in GAD-7 sum score between baseline and the postintervention follow-up in participants with a baseline GAD-7 score ≥5, and change in PHQ-9 sum score between baseline and the postintervention follow-up in participants with a baseline PHQ-9 score ≥5. Secondary analysis will examine differences between baseline and the 3- and 6-month follow-up using a general linear model or a comparable suitable method, including stratification for available baseline characteristics.

#### Secondary Outcomes

Secondary outcomes WSAS and EQ-VAS will be compared between baseline and the 3 primary follow-up time points (4-week, 3-month, and 6-month) in the same manner as the primary outcome.

EQ-5D-5L will be used to determine differences in accrual of QALYs between the trial arms using Norwegian population preference weights. The primary QALY analysis will include only the observed period, with separate analyses using extrapolation to 1- and 3-year time horizons.

The score on the EQ-5D-5L anxiety and depression dimension will be analyzed as a separate measure in the same way as the primary outcome.

The proportion of participants reporting worsened symptoms and treatment-emergent adverse events will also be compared.

#### Health Economic Evaluation

A health economic evaluation will be conducted from the perspective of the public health services. Differences in QALY accrual, as indicated by EQ-5D-5L, will be combined with cost estimates for the apps (no costs assigned to the placebo app) and costs related to reported health services resource use. A standard economic evaluation framework will be used to calculate ICER between the placebo arm and the 2 apps (technically, analyses will compare all 3 apps, as they will be conducted prior to disclosure of which data arm corresponds to which trial arm) [35].

The economic evaluation will follow CHEERS (Consolidated Health Economic Evaluation Reporting Standards) 2022 reporting standards and International Society for Pharmacoeconomics and Outcomes Research (ISPOR) guidelines for economic evaluation of digital health interventions. QALYs will be calculated as the area under the utility curve using linear interpolation between measurement points (baseline, 4 weeks, 3 months, and 6 months), with utilities derived from the Norwegian EQ-5D-5L value set. Unit costs for Norwegian health care services will be sourced from the Norwegian Medical Products Agency. Intervention costs will be estimated as total development and maintenance costs divided by a range of plausible numbers of users completing the app, assuming that the apps would not be substantially revised within the relevant time frame from release. Cost-effectiveness acceptability curves and net health benefit analyses (Stinnett-Mullahy framework) will be used to characterize decision uncertainty under varying willingness-to-pay thresholds.

In addition to the primary economic analysis over the observed 6-month trial period, exploratory extrapolated analyses to 1-year and 3-year time horizons will be conducted using parametric models fit to the observed utility trajectory to inform long-term cost-effectiveness estimates.

### Additional and Exploratory Analyses

Mixed-effects regression models for repeated measures models (mixed model repeated measures) will be used to explore outcomes between trial arms, stratified by demographic characteristics and baseline scores.

Logistic models will be used for binary outcomes and Gaussian models for continuous outcomes.

All analyses will be conducted in R (R Foundation for Statistical Computing). Analysis code will be publicly available with the stage 2 submission as part of the open science commitments of this registered report.

No formal interim efficacy analyses are planned for this trial. The trial may be terminated early if recruitment is insufficient to meet the target sample, subject to agreement from the principal investigator and sponsor (Akershus University Hospital). In the event of an unexpected safety signal, the Trial Oversight Committee/Data Safety Monitoring Board has the authority to recommend early termination.

Considering the brevity of the interventions, no individual discontinuation rules are defined. Patients who report symptoms that would exclude them from participation will be provided the same information on places to seek help as patients excluded (Multimedia Appendix 4).

### Risk Management and Participant Safety

Participants will be screened at baseline for suicidality. Those with severe symptoms or risk will be excluded and directed to appropriate services. The study app will include links to national helplines. Adverse events will be monitored and documented using a structured reporting system within the app.

A data safety monitoring plan will include:

- Weekly review of adverse events by the study team
- Defined escalation procedures for serious adverse events

### User and Stakeholder Involvement

Both Tankevirus and Grubl were codeveloped with lived experience experts, and this project continues that strong commitment to coproduction.

A coresearcher with lived experience of mental health challenges and expertise in digital mental health services is part of the project group and will colead the development of the placebo app. The coresearcher will also participate in a panel with lived experience of mental health challenges. The panel will meet once per project phase (and additionally as needed) and provide structured feedback and advice on:

- Recruitment materials and strategies
- Use of incentives
- Usability and acceptability of both the RCT app and the placebo app
- Interpretation of findings
- Dissemination strategies

Embedding lived experience at multiple levels ensures that the study is ethically sound, scientifically rigorous, and directly relevant to those it seeks to benefit.

### Ethical Considerations

Ethical approval will be sought from the Regional Committee for Medical and Health Research Ethics (Norway) following in-principal approval of the protocol. Participants will provide digital informed consent within the research app.

The trial is sponsored by Akershus University Hospital, Health Services Research Unit, Sykehusveien 25, 1478 Lørenskog, Norway. The sponsor has direct responsibility for the initiation, management, and financing of the trial.

An oversight committee has been formed at the Health Services Research Centre, which will meet regularly during the study. The committee will oversee the project in full and particularly consider any interventions that might be required to ensure participant safety and trial viability.

The Trial Oversight Committee comprises professor HL (chair; head, Health Services Research Centre, Akershus University Hospital), Dr AM (principal investigator), Dr MB, and Dr TW. The Trial Oversight Committee meets quarterly and also functions as the Data Safety Monitoring Board; committee members are independent of the trial sponsor, with the exception of the principal investigator.

The Trial Management Group, responsible for day-to-day trial oversight, comprises: Dr AM (principal investigator, overall responsibility), KR (platform development and statistical analysis), Dr TW (health economic analysis), HHB (intervention development), and JMR-G (study coordination).

The trial will be conducted in accordance with the principles outlined in the Declaration of Helsinki [36] and with Good Clinical Practice. Participants’ anonymity will be maintained throughout.

Any protocol amendments will be submitted to Regional Committee for Medical and Health Research Ethics (Norway) for approval prior to implementation and communicated to the trial registry. Protocol deviations will be recorded in a protocol deviation tracker, reviewed by the oversight committee, and documented in the trial master file.

Data collection and participant engagement are monitored daily on working days. Any significant deviations or potential safety signals identified during daily monitoring are escalated immediately to the oversight committee.

Specific ethical challenges include:

- Design of a placebo condition that is credible yet ethically sound (addressed through provision of general wellness content and posttrial access to active interventions);
- Use of financial incentives to improve adherence (gift cards or vouchers following completion); and
- Handling of personal and sensitive data in accordance with GDPR and Norwegian regulations.

## Results

Funding for this project was approved by the DAM foundation April 28, 2025, conditional on submission of a stage 1 protocol for a registered report by September 15, 2025.

The project timelines, described in a Gannt chart in Multimedia Appendix 5, include submission of the stage 1 protocol in September 2025, followed by finalization of protocol modifications, ethical approval, licensing, and approval of all included instruments from September 2025 through May 2027. Development and testing of Tankevirus version 2.0 is expected to be completed by the end of May 2027. Development and testing of the MIND-APP RCT app will take place from June 2026 to September 2027, alongside final testing from August to November 2026 and pilot testing of the placebo app during the same period. The MIND-APP RCT will launch between September and December 2027, with recruitment and enrollment occurring concurrently. Data collection will run from the start of data collection until 6 months after the final participant has started, no later than August 2028. Analyses are scheduled from August to October 2028, with allocation disclosure in October 2028, followed by final write-up and submission of the stage 2 manuscript toward the end of 2028.

## Discussion

### Principal Findings

This report presents the design and protocol for MIND-APP, a 3-arm RCT evaluating 2 Norwegian self-help smartphone apps; Tankevirus (CBT-based) and Grubl (MCT-based), against a matched digital placebo condition. Registering the protocol prior to data collection ensures analytical transparency and mitigates outcome reporting bias, a recognized strength of the registered report format.

We anticipate that participants in both active arms will show clinically meaningful reductions in anxiety (GAD-7) and depression (PHQ-9) compared with the digital placebo at 4 weeks, with effects potentially sustained at 3- and 6-month follow-up. Given the widespread use of both Tankevirus and Grubl in Norway, with an earlier version of Tankevirus alone reaching approximately 4% of the population, even a modest demonstrated effect would have substantial public health implications. The health economic evaluation will address whether either app represents cost-effective resource use from a Norwegian public health perspective.

### Comparison With Prior Work

Digital mental health apps have shown promise in meta-analytic syntheses. Linardon et al [11] synthesized 176 RCTs and found significant reductions in depression and anxiety, with CBT-based apps showing the most consistent benefits. Lecomte et al [32] found small-to-moderate effects across a meta-review with high heterogeneity. However, most existing studies lack credible active control conditions, making it impossible to disentangle specific therapeutic content effects from nonspecific factors such as digital engagement and expectancy. The matched digital placebo design of MIND-APP directly addresses this gap.

Including a health economic evaluation alongside clinical outcomes is also a distinguishing feature of this trial. By collecting EQ-5D-5L at multiple time points and linking outcomes to self-reported health resource use, MIND-APP will generate incremental cost-effectiveness ratios directly applicable to Norwegian public health policy.

### Strengths and Limitations

Strengths include (1) registered report format with prespecified analyses, (2) large sample (n=1000) providing adequate power for primary comparisons, (3) fully remote design enabling broad population reach within Norway, (4) matched digital placebo controlling for nonspecific effects, (5) integration of clinical and economic outcomes, (6) coproduction with lived experience experts, and (7) comprehensive open science practices.

Limitations include (1) self-reported outcomes only; (2) digital recruitment may favor younger, more digitally literate participants; (3) the placebo, designed to minimize harm, may provide some therapeutic benefit, attenuating effect sizes; (4) Norwegian-only restriction limits generalizability; (5) not powered for direct Tankevirus vs Grubl comparison; and (6) 6-month follow-up does not capture long-term durability.

### Conclusions

MIND-APP will provide the first rigorously controlled clinical and economic evaluation of 2 of Norway’s most widely used mental health apps. By using a registered report format, a matched digital placebo, and a comprehensive outcomes battery including QALYs and health resource use, this trial is designed to generate evidence informing clinical practice and public health policy. Regardless of findings, results will be published, contributing to the cumulative evidence base on digital mental health interventions.

Results will be disseminated in open-access peer-reviewed format regardless of findings. Authorship will follow International Committee of Medical Journal Editors (ICMJE) criteria. Norwegian-language summaries for clinical and patient audiences are planned following peer-reviewed publication.

## Data Availability

Fully anonymized participant-level data, a complete data dictionary, and all analysis code (R) will be deposited in a public repository (Open Science Framework [OSF] or equivalent) at the time of Stage 2 publication. Directly identifying information will be deleted prior to final analysis and will not be shared.

## Appendix

Multimedia Appendix 1 SPIRIT 2025 checklist.

Multimedia Appendix 2 Detailed description of the Placebo app (currently a placeholder).

Multimedia Appendix 3 List of self-help applications to be asked for at screening.

Multimedia Appendix 4 Information for patients ineligible due to severe anxiety, depression, or suicidal ideation.

Multimedia Appendix 5 Gannt chart.

## Abbreviations

ANCOVA: analysis of covariance
CBT: cognitive behavioral therapy
CHEERS: Consolidated Health Economic Evaluation Reporting Standards
CSQ-8: Client Satisfaction Questionnaire-8
DSM-IV: Diagnostic and Statistical Manual of Mental Disorders (Fourth Edition)
EQ-VAS: EQ visual analog scale
GAD-7: Generalized Anxiety Disorder-7
GDPR: General Data Protection Regulation
HRQoL: health-related quality of life
ICER: incremental cost-effectiveness ratio
ICMJE: International Committee of Medical Journal Editors
ISPOR: International Society for Pharmacoeconomics and Outcomes Research
MCT: metacognitive therapy
MIND-APP: Mental Health Intervention With Digital Applications
PHQ-4: Patient Health Questionnaire-4
PHQ-9: Patient Health Questionnaire-9
QALY: quality-adjusted life year
RCT: randomized controlled trial
SPIRIT: Standard Protocol Items: Recommendations for Interventional Trials
SSS-8: Somatic Symptom Scale 8-item
SUS: System Usability Scale
WHO: World Health Organization
WSAS: Work and Social Adjustment Scale

## References

[1] Chisholm D, Sweeny K, Sheehan P, Rasmussen B, Smit F, Cuijpers P, Saxena S (2016). Scaling-up treatment of depression and anxiety: a global return on investment analysis. Lancet Psychiatry.

[2] Sandanger I, Nygård JF, Ingebrigtsen G, Sørensen T, Dalgard OS (1999). Prevalence, incidence and age at onset of psychiatric disorders in Norway. Soc Psychiatry Psychiatr Epidemiol.

[3] Nystuen P, Hagen KB, Herrin J (2001). Mental health problems as a cause of long-term sick leave in the Norwegian workforce. Scand J Public Health.

[4] Mykletun A, Overland S, Dahl AA, Krokstad S, Bjerkeset O, Glozier N, Aarø LE, Prince M (2006). A population-based cohort study of the effect of common mental disorders on disability pension awards. Am J Psychiatry.

[5] Knapstad M, Sæther SMM, Hensing G, Smith O (2020). Prompt mental health care (PMHC): work participation and functional status at 12 months post-treatment. BMC Health Serv Res.

[6] Arias D, Saxena S, Verguet S (2022). Quantifying the global burden of mental disorders and their economic value. EClinicalMedicine.

[7] Smit HFE, Cuijpers P, Oostenbrink J, Batelaan NM, de Graaf R, Beekman A (2006). Costs of nine common mental disorders: implications for curative and preventive psychiatry. J Ment Health Policy Econ.

[8] Norwegian Ministry of Health and Care Services (2023). Escalation Plan for Mental Health (2023–2033): Report to the Storting (White Paper).

[9] Neary M, Schueller S (2018). State of the field of mental health apps. Cogn Behav Pract.

[10] World Health Organization (2021). Comprehensive Mental Health Action Plan 2013-2030.

[11] Linardon J, Torous J, Firth J, Cuijpers P, Messer M, Fuller-Tyszkiewicz M (2024). Current evidence on the efficacy of mental health smartphone apps for symptoms of depression and anxiety. A meta-analysis of 176 randomized controlled trials. World Psychiatry.

[12] Furukawa TA, Noma H, Caldwell DM, Honyashiki M, Shinohara K, Imai H (2014). Waiting list may be a nocebo condition in psychotherapy trials: a contribution from network meta-analysis. Acta Psychiatr Scand.

[13] Uwatoko T, Luo Y, Sakata M, Kobayashi D, Sakagami Y, Takemoto K (2018). Healthy campus trial: a multiphase optimization strategy (MOST) fully factorial trial to optimize the smartphone cognitive behavioral therapy (CBT) app for mental health promotion among university students: study protocol for a randomized controlled trial. Trials.

[14] Smith ME, Sharma D, Rivero-Arias O, Rand K, Barrack L, Ogburn E (2024). Digital thErapy for improved tiNnitus carE study (DEFINE): protocol for a randomised controlled trial. PLoS One.

[15] Pocock SJ, Simon R (1975). Sequential treatment assignment with balancing for prognostic factors in the controlled clinical trial. Biometrics.

[16] Wells A (1990). Panic disorder in association with relaxation induced anxiety: an attentional training approach to treatment. Behav Ther.

[17] Dammen T, Tunheim K, Munkhaugen J, Papageorgiou C (2022). The attention training technique reduces anxiety and depression in patients with coronary heart disease: a pilot feasibility study. Front Psychol.

[18] Fergus TA, Bardeen JR (2016). The attention training technique: a review of a neurobehavioral therapy for emotional disorders. Cogn Behav Pract.

[19] Spitzer RL, Kroenke K, Williams JBW, Löwe B (2006). A brief measure for assessing generalized anxiety disorder: the GAD-7. Arch Intern Med.

[20] Kroenke K, Spitzer RL, Williams JB, Monahan PO, Löwe B (2007). Anxiety disorders in primary care: prevalence, impairment, comorbidity, and detection. Ann Intern Med.

[21] Toussaint A, Hüsing P, Gumz A, Wingenfeld K, Härter M, Schramm E, Löwe B (2020). Sensitivity to change and minimal clinically important difference of the 7-item generalized anxiety disorder questionnaire (GAD-7). J Affect Disord.

[22] Kroenke K, Spitzer RL, Williams JBW (2001). The PHQ-9: validity of a brief depression severity measure. J Gen Intern Med.

[23] Löwe B, Kroenke K, Herzog W, Gräfe K (2004). Measuring depression outcome with a brief self-report instrument: sensitivity to change of the patient health questionnaire (PHQ-9). J Affect Disord.

[24] Manea L, Gilbody S, McMillan D (2012). Optimal cut-off score for diagnosing depression with the patient health questionnaire (PHQ-9): a meta-analysis. CMAJ.

[25] Herdman M, Gudex C, Lloyd A, Janssen M, Kind P, Parkin D, Bonsel G, Badia X (2011). Development and preliminary testing of the new five-level version of EQ-5D (EQ-5D-5L). Qual Life Res.

[26] Wisløff T, Hagen G, Hamidi V, Movik E, Klemp M, Olsen JA (2014). Estimating QALY gains in applied studies: a review of cost-utility analyses published in 2010. Pharmacoeconomics.

[27] Garratt AM, Stavem K, Shaw JW, Rand K (2025). EQ-5D-5L value set for Norway: a hybrid model using cTTO and DCE data. Qual Life Res.

[28] Mundt J, Marks I, Shear M, Greist J (2002). The work and social adjustment scale: a simple measure of impairment in functioning. Br J Psychiatry.

[29] Attkisson CC, Larsen D, Hargreaves WA, LeVois M, Nguyen R, Stegner B (1987). Client Satisfaction Questionnaire (CSQ-8). Measures for Clinical Practice: A Sourcebook.

[30] Brooke J (1996). SUS-A quick and dirty usability scale. Usability Evaluation in Industry.

[31] Bangor A, Kortum PT, Miller JT (2008). An empirical evaluation of the system usability scale. Int J Hum Comput Interact.

[32] Lecomte T, Potvin S, Corbière M, Guay S, Samson C, Cloutier B (2020). Mobile apps for mental health issues: meta-review of meta-analyses. JMIR Mhealth Uhealth.

[33] Hooper R (2025). To adjust, or not to adjust, for multiple comparisons. J Clin Epidemiol.

[34] Dunnett CW (1955). A multiple comparison procedure for comparing several treatments with a control. J Am Stat Assoc.

[35] Drummond MF, Schulpher MJ, Claxton K, Stoddart GL, Torrance GW (2015). Methods for the Economic Evaluation of Health Care Programmes.

[36] World Medical Association (2025). World Medical Association Declaration of Helsinki: ethical principles for medical research involving human participants. JAMA.

[37] GAIDeT (Generative AI Delegation Taxonomy): A taxonomy for humans to delegate tasks to generative artificial intelligence in scientific research and publishing: Accountability in Research.

