# Supplementary material for: Effectiveness and Cost-Effectiveness of the Tankevirus and Grubl Mental Health Apps Compared With a Placebo App—Mental Health Intervention With Digital Applications (MIND-APP): Protocol for a Randomized Controlled Researcher-Blinded Trial
Source: JMIR Res Protoc. 2026 Aug 10;15:e84096. doi: 10.2196/84096 (PMC13504246; doi:10.2196/84096)
Supplement: Supplementary file 1 [file resprot_v15i1e84096_app1.docx]

# Appendix 3: SPIRIT 2025 checklist

**SPIRIT 2025 Checklist — MIND-APP Protocol**

Revision for JMIR Research Protocols resubmission. Updated 2026-04-10.

Status key: OK = adequately addressed; ADDED = added in revision; PARTIAL = present but incomplete; MISSING = absent; N/A = not applicable.

Items with status MISSING or PARTIAL require author action before submission.

| **Item No.** | **Topic** | **Status** | **Notes / Action Required** |
| --- | --- | --- | --- |
| **ADMINISTRATIVE INFORMATION** | | | |
| 1a | Title (design, population, intervention, "protocol") | PARTIAL | Title includes design elements, population, intervention, and 'Protocol'. However, title format should follow JMIR guidelines: 'Intervention in Condition: Study Design'. JMIR research protocols only allows registered report protocols, so this is implied. |
| 1b | Structured summary | OK | Abstract structured with Background/Objectives/Methods/Results/Conclusions headers. |
| Proto Ver | Protocol version / date | OK | Version 1.0 and April 10^th^ 2026 stated. |
| 3a | Names, affiliations, roles of contributors | OK | Author block with ORCIDs + Authors' Contributions section using CRediT taxonomy. |
| 3b | Sponsor name and contact | OK | Akershus University Hospital, Health Services Research Unit, Sykehusveien 25, 1478 Lorenskog. |
| 3c | Role of sponsor and funders | OK | Sponsor role (initiation, management, financing) stated. DAM Foundation no-interference statement in Acknowledgments. |
| 3d | Steering committee / DSMB composition and independence | OK | TOC: Prof. Hilde Luras (Chair), Dr Angela Menichetti (PI), Dr Mathias Barra, Dr Torbjorn Wisloff. Independence statement present. |
| TMG | Trial Management Group | OK | TMG: Dr Angela Menichetti (PI), Kim Rand, Dr Torbjorn Wisloff, Hanne H. Brorson, Juan Manuel Ramos-Goni. |
| 5 | Trial registration (name, ID, URL, date) | MISSING | XXXX placeholder remains for ClinicalTrials.gov. |
| 6 | Protocol and SAP location | OK | The manuscript is the full protocol. SPIRIT checklist as Appendix 3. |
| 7a | Funding sources | OK | DAM Foundation, project ID 26FORS701644 RR, in Acknowledgments. |
| 7b | Conflicts of interest | OK | COI section present. Brorson (IP owner), Rand (co-founder Maths in Health) disclosed. |
| 8 | Dissemination policy | OK | Open-access publication, ICMJE authorship, Norwegian-language summaries planned. |
| **INTRODUCTION** | | | |
| 9a | Scientific background and rationale | OK | Expanded: CBT/MCT theoretical frameworks, digital placebo concept, cost-effectiveness gap. MCT attention training evidence added with new references [16-18]. |
| 9b | Choice of comparator — rationale | OK | Digital placebo rationale explained. Waitlist nocebo concern cited [12]. Three-arm rationale paragraph present. |
| 10 | Specific objectives | OK | Co-primary: anxiety reduction (GAD-7) and depression reduction (PHQ-9). HRQoL, QALYs, ICER) objectives stated. Hypotheses clearly formulated. |
| **METHODS: TRIAL DESIGN** | | | |
| 11 | Patient and public involvement | OK | User and Stakeholder Involvement section: co-researcher with lived experience, user panel meeting per project phase. |
| 12 | Trial design (type, allocation ratio, framework) | OK | Three-arm parallel 1:1:1 RCT, open-label, analyst-blinded, superiority framework. |
| **METHODS: PARTICIPANTS, INTERVENTIONS, OUTCOMES** | | | |
| 13 | Settings and locations | OK | Fully remote via bespoke smartphone app. Norway. iOS and Android. |
| 14a | Eligibility criteria for participants | OK | Inclusion: age >=16, Norway, smartphone, PHQ-9/GAD-7 >=5. Exclusion: PHQ-9 >=20, suicidal ideation, hospitalization, psychotropic medications, current therapy, prior app use. |
| 14b | Eligibility criteria for sites/deliverers | N/A | Fully automated; no investigator-dependent enrolment. |
| 15a | Intervention details (sufficient to replicate) | OK | Tankevirus (CBT), Grubl (MCT), and digital placebo described. Shared characteristics, intervention duration, and content principles stated. Figures 1-3 provide visual examples. |
| 15b | Criteria for discontinuing intervention | PARTIAL | PHQ-9 >=20 screening exclusion and in-app symptom monitoring present. No formal mid-study individual discontinuation criterion stated. Trial-level stopping rules present (insufficient recruitment, safety signal). |
| 15c | Adherence strategies and monitoring | OK | Per-protocol definition (3 primary videos/units completed). Daily push notifications. Financial incentives (NOK 500). 2-week lost-to-follow-up rule. |
| 15d | Concomitant care (permitted or prohibited) | OK | Self-help apps prohibited during 4-week intervention. Therapy prohibited during intervention, permitted during follow-up. No medications prohibited. Concurrent treatment assessed at each follow-up. |
| 16 | Primary and secondary outcomes | OK | Co-primary: GAD-7 and PHQ-9 change at 4 weeks. Secondary: GAD-7/PHQ-9 at 3/6 months, EQ-5D-5L, QALYs, WSAS, health resource use. CSQ-8 and SUS listed as 'tertiary'. |
| 17 | Harms — definition and assessment method | OK | AE/SAE definitions per ICH. Causality levels (unrelated/possibly/probably/definitely). In-app reporting. Weekly AE review by study team. List of anticipated AEs. |
| 18 | Participant timeline (schedule of events) | OK | Table 1 includes all assessments: PHQ-9, GAD-7, EQ-5D-5L, WSAS, CSQ-8, SUS, co-treatment check, resource use at appropriate timepoints. |
| 19 | Sample size | OK | 300/arm from Monte Carlo simulation. Cohen's d = 0.3, 80% power, alpha = 0.05. Correlation r = 0.7. Recruitment target 1000 for attrition. |
| 20 | Recruitment strategies | OK | Social media, university health services, primary care (informational materials only; no active GP screening/referral), mental health organizations, patient communities. |
| **METHODS: ASSIGNMENT OF INTERVENTIONS** | | | |
| 21a | Randomization sequence generation method | OK | Pocock-Simon covariate-adaptive minimization with biased coin [15]. 100-participant burn-in with simple equal-probability assignment. |
| 21b | Type of randomization and stratification factors | OK | 1:1:1 stratified by sex, age group (18-30 / 31-50 / 51+), and baseline severity (above/below GAD-7 or PHQ-9 score 10). |
| 22 | Allocation concealment mechanism | OK | Centralized platform. Arm assignments in separate database partition, inaccessible to study team prior to data lock. |
| 23 | Personnel for enrolment vs. assignment | OK | Fully automated; no investigator manually assigns participants. |
| 24a | Who is blinded | OK | Participants not blinded (branded apps). Research team analyst-blinded. |
| 24b | How blinding is maintained | OK | Arm identifiers removed from analysis dataset until analytical lock. Unblinding by platform administrator upon confirmation of lock. |
| 24c | Unblinding circumstances | N/A | Open-label to participants; analyst-blinding only. |
| **METHODS: DATA COLLECTION, MANAGEMENT, AND ANALYSIS** | | | |
| 25a | Data collection methods; instrument reliability/validity | PARTIAL | All instruments described with scoring and cut-offs. GAD-7 reliability reported (Cronbach alpha >= 0.92). PHQ-9 and WSAS reliability statistics not provided. |
| 25b | Retention and complete follow-up | OK | Push notification reminders. 2-week lost-to-follow-up rule at all timepoints. |
| 26 | Data management (entry, coding, security, storage) | OK | VPN/firewall, username/password/certificate, audit trail. Identifiable data stored separately, encrypted, GDPR compliant. Deleted upon data collection completion. |
| 27a | Statistical methods for primary/secondary outcomes | OK | ANCOVA with baseline covariate + stratification variables. Dunnett's procedure for two treatment-vs-placebo comparisons [34]. MMRM for repeated measures. Health economic evaluation (CHEERS/ISPOR). |
| 27b | Analysis population (ITT, per-protocol) | OK | Per-protocol primary analysis. ITT with imputed values as sensitivity analysis. |
| 27c | Missing data handling | OK | MMRM under MAR (maximum likelihood). Multiple imputation if evidence of MNAR. |
| 27d | Subgroup/sensitivity analyses | OK | MMRM exploratory analyses stratified by demographics and baseline scores. Logistic models for binary outcomes, Gaussian for continuous. |
| **METHODS: MONITORING** | | | |
| 28a | Data monitoring committee (composition, role, independence) | OK | TOC functions as DSMB. Named members. Independence statement present (exception: PI is a member). |
| 28b | Interim analyses and stopping guidelines | OK | No formal interim efficacy analyses. Early termination for insufficient recruitment or unexpected safety signal (TOC/DSMB authority). |
| 29 | Trial monitoring (frequency and procedures) | OK | Daily monitoring on working days. Weekly AE review. Quarterly REK reports. Immediate escalation for deviations/safety signals. |
| **ETHICS** | | | |
| 30 | Ethics approval | PARTIAL | REK Norway approval to be sought following in-principle acceptance of protocol. No case number yet. |
| 31 | Protocol amendments | OK | REK approval before implementation. Registry notification. Protocol Deviation Tracker. Documented in trial master file. |
| 32a | Informed consent | OK | Digital consent within app. Name, age, phone number collected. SMS verification. Age >= 16 (ethical review for under-18s noted). |
| 32b | Ancillary studies — consent | N/A | No ancillary studies planned. |
| 33 | Confidentiality | OK | GDPR/Norwegian Personal Data Act compliant. VPN/firewall. Identifiable data stored separately, encrypted, deleted upon completion. 7-month deletion window for withdrawal requests. |
| 34 | Ancillary and post-trial care / compensation for harm | OK | Post-trial: all participants offered active apps. Compensation: NOK 500 (one-off or two tranches of NOK 250). Charitable donation alternative offered. |
