# Supplementary material for: Effectiveness and Cost-Effectiveness of the Tankevirus and Grubl Mental Health Apps Compared With a Placebo App—Mental Health Intervention With Digital Applications (MIND-APP): Protocol for a Randomized Controlled Researcher-Blinded Trial
Source: JMIR Res Protoc. 2026 Aug 10;15:e84096. doi: 10.2196/84096 (PMC13504246; doi:10.2196/84096)
Supplement: Supplementary file 3 [file resprot_v15i1e84096_app3.docx]

# Appendix 2: List of self-help applications to be asked for at screening

- Headspace – mindfulness
- Calm – mindfulness, sleep, breathing
- Happify – CBT/positive psychology
- Tankevirus – CBT, metaphors, videos, reflection
- Grubl – MCT, reflection exercises, activities
- Assistert selvhjelp – CBT modules
- Overvinne – self-help programs
- Selvhjelperen – CBT app
- Pustepause – mindfulness exercises
- Mindfulness-appen – guided meditation
- Abel – AI-supported self-work
- UngSpotlight – for youth
- Reflectly – journaling + reflection
- Daylio – mood diary / mood tracking
- Finch: Self-Care Pet – gamified self-care
- Moodfit – tracking + exercises
- Happier meditation – guided meditation
