# Supplementary material for: Effectiveness and Cost-Effectiveness of the Tankevirus and Grubl Mental Health Apps Compared With a Placebo App—Mental Health Intervention With Digital Applications (MIND-APP): Protocol for a Randomized Controlled Researcher-Blinded Trial
Source: JMIR Res Protoc. 2026 Aug 10;15:e84096. doi: 10.2196/84096 (PMC13504246; doi:10.2196/84096)
Supplement: Supplementary file 4 [file resprot_v15i1e84096_app4.docx]

# Appendix 1: Information to patients ineligible due to severe anxiety, depression, or suicidal ideation

Participants indicating severe depression (PHQ-9 ≥ 20) or a score of 2 or higher on PHQ-9 item 9, suggesting suicidal ideation, are informed that they are not eligible to participate in the trial. They are provided with the following information, here in English, on where to seek help in Norway:

- In case of emergency: Call 113 for immediate medical assistance.
- Mental Health Helpline (Mental Helse Hjelpetelefonen): Call 116 123 for free, anonymous support 24/7.
- Kirkens SOS: Call 22 40 00 40 or chat online at [URL] for confidential conversations 24/7.
- Contact your general practitioner (fastlege) to discuss your mental health and treatment options.
- Additional resources and information are available at helsenorge.no and mentalhelse.no.

Please seek help if you are experiencing distress—support is available, and you do not have to face this alone.
