# Supplementary material for: Effectiveness and Cost-Effectiveness of the Tankevirus and Grubl Mental Health Apps Compared With a Placebo App—Mental Health Intervention With Digital Applications (MIND-APP): Protocol for a Randomized Controlled Researcher-Blinded Trial
Source: JMIR Res Protoc. 2026 Aug 10;15:e84096. doi: 10.2196/84096 (PMC13504246; doi:10.2196/84096)
Supplement: Supplementary file 5 [file resprot_v15i1e84096_app2.docx]

Appendix 2: Detailed description of the Placebo app. PLACEHOLDER
